# Attenuated Seasonality of Childhood Type 1 Diabetes with Increasing Autoimmune Comorbidities: A 40-year Cohort Study

**DOI:** 10.1007/s11845-026-04558-x

**Published:** 2026-07-22

**Authors:** Gulsum Ozen, Angela Zanfardino, Gulsah Ozen Acan, Burak Acan, Nadia Tinto, Fernanda Iafusco, Emanuele Miraglia del Giudice, Zehra Aycan, Dario Iafusco

**Affiliations:** 1 Regional Centre for Pediatric Diabetes, Department of Pediatrics, University of Campania “Luigi Vanvitelli”, Naples, Italy; 2 Department of Pediatrics- Adolescent Health, Faculty of Medicine, Ankara University, Ankara, Turkey; 3 Department of Pediatrics, Ankara Ataturk Sanatoryum Training and Research Hospital, Ankara, Turkey; 4 Department of Pediatric and Adolescent Psychiatry, Faculty of Medicine, Hacettepe University, Ankara, Turkey; 5 Department of Emergency, Etimesgut Şehit Sait Ertürk State Hospital, Ankara, Turkey; 6 CEINGE- Advanced Biotechnologies Franco Salvatore, University ‘‘Federico II’’, Naples, Italy; 7 Department of Pediatric Endocrinology, Faculty of Medicine, Ankara University, Ankara, Turkey

**Keywords:** Autoimmunity, Celiac Disease, Diabetes Mellitus, Environmental factors, Hashimoto’s Thyroiditis, Seasonality

## Abstract

**Background:**

Seasonal variation in the onset of Type 1 Diabetes Mellitus (T1DM) has long been recognized, yet little is known about how the *severity of autoimmunity* interacts with environmental triggers to influence disease manifestation.

**Aims:**

This study aimed to elucidate the etiological interplay between seasonality, genetic predisposition, and immune dysregulation over a 40-year period.

**Methods:**

A retrospective cohort of 2,954 patients diagnosed with T1DM between 1981 and 2024 was analyzed. Seasonal trends in disease onset and autoimmune comorbidities were evaluated acoording to age at onset, birth season, and sex.

**Results:**

Distinct age and immunity-dependent seasonal patterns were identified. Early-onset T1DM showed no seasonal fluctuation, suggesting predominant genetic and immunological determinants. Conversely, late-onset T1DM demonstrated a clear autumn–winter peak and was more frequent among children born in spring–summer. The presence of multiple autoimmune disorders abolished this seasonality, implying that intense autoimmune activity may override environmental effects.

**Conclusions:**

This 40-year cohort provides novel evidence that the relative contribution of environmental and immunogenetic factors in T1DM varies with age and immune burden. Environmental seasonality appears to play a greater role in later-onset disease, whereas early-onset and multi-autoimmune forms are driven by intrinsic immune mechanisms. These findings highlight the heterogeneity of T1DM pathogenesis and support the need for age- and immunity-specific strategies for prevention and early screening.

## Introduction

Type 1 Diabetes Mellitus (T1DM) is a chronic, autoimmune, multifactorial disease, usually with childhood-onset. It characterized by immune-mediated destruction of pancreatic β-cells in genetically susceptible individuals, insulin deficiency, and resultant hyperglycemia [1]. Although its etiology has not been fully elucidated and genetic predisposition is essential, environmental factors are believed to play a role in the initiation and progression of the disease [2]. Evidence suggests that infections, dietary components, toxins, and hygiene-related factors can initiate or accelerate autoimmunity in genetically susceptible individuals. The first peak of the diagnosis of T1DM occurs in children aged 5–7 years which is attributed to increased exposure to infections upon starting school [3].

Environmental factors are thought to contribute to β-cell destruction by triggering autoimmune responses. Seasonal fluctuations in disease onset have been observed in many high-incidence countries such as Finland and Sweden, with peaks in winter and autumn months [4–6]. However, findings from low-incidence regions such as China and Japan have shown inconsistent patterns, suggesting that environmental and immunological factors interact in complex ways across populations [7–9].

Most previous studies have evaluated T1DM seasonality according to age, sex or geographic region but rarely considered autoimmune comorbidity that may reflect the intensity of immune dysregulation [7]. Because the coexistence of additional autoimmune diseases may reflect a stronger genetic and immunological predisposition, the contribution of environmental factors to disease onset may differ in these individuals. Consequently, seasonality may become less pronounced as autoimmune disease burden increases, reflecting a shift from environmental toward genetic and immunological determinants.

Based on this hypothesis, we investigated whether the seasonal patterns of birth and T1DM diagnosis differ according to the presence and number of associated autoimmune diseases, including celiac disease and Hashimoto’s thyroiditis, while also evaluating the effect of age at disease onset. We hypothesized that seasonal variation would be attenuated in children with multiple autoimmune diseases and in those diagnosed at an earlier age, suggesting a greater contribution of genetic and immunological factors compared with environmental influences. By integrating environmental, developmental, and immunological perspectives, this work seeks to refine our understanding of T1DM etiology and guide future prevention strategies.

## Materials and methods

### Study design

This retrospective cohort analysis included all patients diagnosed with T1DM before 18 years of age between 1981 and 2024 at our diabetology center. Diagnosis followed the International Society for Pediatric and Adolescent Diabetes (ISPAD) current diagnostic criteria. Data were extracted from the electronic health information system ‘Aretheus’.

Patients were excluded if data were incomplete, diagnostic criteria were not met, or diagnoses changed during follow-up (e.g., Type 2 DM or Maturity-Onset Diabetes of the Young (MODY) misclassification).

### Definitions

To account for potential etiological-immunological differences between early and late-onset diseases, patients were divided by age at onset: early-onset (< 3 years) vs. late-onset (≥ 3 years). This threshold was selected based on internationally accepted definitions of early childhood education and daycare attendance. Before the age of 3 years, children are predominantly cared for at home and generally have lower exposure to daycare, preschool settings, peer contact, and community-acquired infectious agents. Accordingly, the age of 3 years has been widely adopted in the literature as the transition point between early daycare attendance and preschool exposure [10, 11]. Furthermore, the OECD Family Database identifies the third birthday as the breakpoint because children become eligible for pre-primary education in most OECD countries at this age. Although enrollment of children younger than 3 years in daycare has increased in recent years, the 3-year threshold remains the internationally accepted standard for distinguishing the external exposure periods [12]. Patients were also divided by autoimmune status: T1DM only; T1DM + CD; T1DM + HT; T1DM + CD + HT to evaluate intensity of immune dysregulation [13, 14].

Hashimoto’s thyroiditis was defined by physical examination, elevated thyroid stimulating hormone (*TSH)* levels, and thyroid antibodies, confirmed by ultrasonography. Celiac disease was diagnosed via elevated levels of specific antibodies (immunoglobulin A anti-tissue transglutaminase antibody (anti-tTG-IgA), immunoglobulin A anti-endomysial antibodies (EMA-IgA) and biopsy-confirmed enteropathy in patients presenting with typical signs and symptoms such as diarrhea and malabsorption.

Seasons were classified as: spring (March–May), summer (June–August), autumn (September–November), and winter (December-February). We evaluated the seasonal incidence of T1DM, CD, and HT in all individuals. Data on the two most common autoimmune diseases associated with T1DM (CD, HT) were recorded. In addition to the season of diagnosis, we also examined the relationship between birth season and gender in these autoimmune diseases. An additional comparison was made between the autumn–winter months, when both respiratory and gastrointestinal infections are more common, and the spring–summer months. The relationship between these variables and age at diagnosis was also assessed. In this way, we evaluated the differences between early-onset and late-onset diseases in terms of etiology and immune response.

### Statistical analysis

Data were analyzed by using the IBM Statistical Package for Social Sciences (IBM-SPSS Statistics, 22). The Kolmogorov–Smirnov test was applied to assess the normality of data distribution. Chi-square tests were used to compare the differences in the frequencies of categorical groups. The independent samples t-test was performed to compare the means of two independent groups with normal distribution and Mann–Whitney U test for non-normally distributed data. Correlation analysis of non-normally distributed values was performed by Spearman’s correlation, while Pearson’s correlation was used for normally distributed values. Multivariable binary logistic regression analyses were conducted separately according to age at diabetes onset (< 3 years and ≥ 3 years), and results were expressed as odds ratios (ORs) with 95% confidence intervals (CIs). Statistical significance was set at a p value of < 0.05 (*p* < 0.05).

### Ethics

This study was approved by the local ethics committee of the University (N prot:694 12.11.19) and was conducted in accordance with the Declaration of Helsinki. The authors declare they have no conflicts of interest. This research received no external funding.

## Results

### Demographic and clinical characteristics

The cohort included 2954 patients (45.9% female, mean age 23.4 ± 9.7 years). The demographic characteristics of the study population are shown in Table 1. A significant male predominance was observed in late-onset T1DM (*p* < 0.005). Early-onset cases showed no gender difference (*p* = 0.431).

**Table 1:** Demographic characteristics of the study population

	General population	Female	Male	*p* value
Number of patients (n,%) ^a^	2954	1357 (45.9%)	1597 (54.1%)	** < 0.001**
Age (years) ^b^	23.36 ± 9.72	23.22 ± 9.74	23.49 ± 9.69	0.449
Autoimmune diseases (n, %) ^a^				
Only DM	2249 (76.1%)	950 (70%)	1299 (81.3%)	** < 0.001**
DM + only CD	184 (6.2%)	82(6.1%)	102 (6.4%)	0.127
DM + only HT	462 (15.6%)	284 (20.9%)	178 (11.2%)	** < 0.001**
DM + CD + HT	59 (2%)	41 (%3)	18 (1.1%)	** < 0.001**
Age at the onset of DM (years) (min–max) ^b^	8.66 ± 4.14 (6 months- 18 years)	8.37 ± 4.07 (5 months-18 years)	8.91 ± 4.18 (5 months- 18 years)	** < 0.001**
< 3 years old at the onset of DM (n, %) ^a^	316/2954 (10.7%)	165	151	
Only DM	206 (65.2%)	96 (58.2%)	110 (72.8%)	**0.006**
DM + only CD	42 (13.3%)	19 (11.5%)	23 (15.2%)	0.744
DM + only HT	49 (15.5%)	36 (21.8%)	13 (8.6%)	** < 0.001**
DM + CD + HT	19 (6%)	14 (8.5%)	5 (3.3%)	0.054
≥ 3 years old at the onset of DM (n, %) ^a^	2638/2954 (89.3%)	1192	1446	
Only DM	2043 (77.4%)	854(71.6%)	1189 (82.2%)	** < 0.001**
DM + only CD	142 (5.4%)	63 (5.3%)	79 (5.5%)	0.231
DM + only HT	413 (15.7%)	248(20.8%)	165 (11.4%)	** < 0.001**
DM + CD + HT	40 (1.5%)	27 (2.3%)	13 (0.9%)	**0.004**
Season at onset of DM (n, %) ^a^				0.142
Spring	729 (24.7%)	327 (24.1%)	402(25.2%)	
Summer	562 (19%)	276 (20.3%)	286(17.9%)	
Autumn	807(27.3%)	377 (27.8%)	430(26.9%)	
Winter	856 (29%)	377 (27.8%)	479(30%)	
Birth season (n, %) ^a^				0.541
Spring	726 (24.6%)	341 (25.1%)	385(24.1%)	
Summer	825 (27.9%)	381 (28.1%)	444(27.8%)	
Autumn	729 (24.7%)	325 (23.9%)	404(25.3%)	
Winter	674 (22.8%)	310 (22.8%)	364(22.8%)	

*n* number, *min–max* minimum–maximum, *DM* Diabetes Mellitus, *HT* Hashimoto thyroiditis, *CD* Celiac disease. Continuous variables were expressed as mean ± standard deviation (SD), while categorical variables were presented as frequencies and within-group percentages. mean ± SD, n (%). ^*a*^* p* < *.05 assessed through ****chi-squared analyses ***^***b***^* p* < *.05 assessed through ****independent samples t- analysis***

Coexisting autoimmune diseases were significantly more prevalent among early-onset T1DM patients (*p* = 0.002), particularly CD (*p* < 0.001). The youngest mean age at onset occurred in patients with both CD and HT (*p* = 0.004) (Table 2).

**Table 2:** Seasonal variations of Type 1 DM and other autoimmune diseases

General population		Only DM (2249)		DM + CD (243)		DM + HT (521)		DM + CD + HT(59)	
	Gender ^*a*^ n(%)								
	Female	950(70%)		123(9%)		325(24%)		41(3%)	
	Male	1299(81.3%)		120(7.5%)		196(12.2%)		18 (1.1%)	
	*p* value	< 0.001		0.847		< 0.001		0.003	
	Age at onset of DM (years)^*b*^	Only DM	8.79 ± 4.10	DM + CD	6.97 ± 4.35	DM + HT	8.68 ± 4.14	DM + CD + HT	6.88 ± 4.66
		Others	8.24 ± 4.23	Others	8.81 ± 4.09	Others	8.66 ± 4.14	Others	8.70 ± 4.12
	*p* value	0.002		< 0.001		0.925		0.004	
	Season at birth^*a*^								
	Spring	538(23.9%)		59(24.3%)		145(27.8%)		16(27.1%)	
	Summer	642(28.5%)		69(28.4%)		126(24.2%)		12(20.3%)	
	Autumn	550(24.5%)		66(27.2%)		129(24.8%)		16(27.1%)	
	Winter	519 (23.1%)		49(20.1%)		121(23.2%)		15(25.4%)	
	*p* value	0.001		0.273		0.479		0.866	
	Season at onset of DM^*a*^								
	Spring	558(24.8%)		52(21.4%)		135(25.9%)		16(27.1%)	
	Summer	417(18.5%)		52(21.4%)		105(20.2%)		12(20.3%)	
	Autumn	601(26.7%)		69(28.4%)		157 (30.1%)		20(33.9%)	
	Winter	673(29.9%)		70(28.8%)		124(23.8%)		11(18.6%)	
	*p* value	< 0.001		0.025		0.012		0.329	
Age at onset < 3	Season at birth^*a*^	Only DM (206)		DM + CD (61)		DM + HT (68)		DM + CD + HT(19)	
	Spring	52(25.2%)		12(19.7%)		18(26.5%)		5(26.3%)	
	Summer	64(31.1%)		18(29.5%)		22(32.4%)		5(26.3%)	
	Autumn	51(24.8%)		16(26.2%)		11(16.2%)		3(15.8%)	
	Winter	39(18.9%)		15(24.6%)		17(25%)		6(31.6%)	
	*p* value	0.108		0.746		0.302		0.801	
	Season at onset of DM^*a*^								
	Spring	52(25.2%)		10(16.4%)		21(30.9%)		5 (26.3%)	
	Summer	53(25.7%)		18(29.5%)		16(23.5%)		3(15.8%)	
	Autumn	59(28.6%)		20(32.8%)		15(22.1%)		8(42.1%)	
	Winter	42(20.4%)		13(21.3%)		16(23.5%)		3(15.8%)	
	*p* value	0.408		0.249		0.731		0.317	
Age at onset ≥ 3	Season at birth^*a*^	Only DM (2043)		DM + CD (182)		DM + HT (453)		DM + CD + HT(40)	
	Spring	486(23.8%)		47(25.8%)		127(28%)		11(27.5%)	
	Summer	578(28.3%)		51(28%)		104(23%)		7(17.5%)	
	Autumn	499(24.4%)		50(27.5%)		118(26%)		13(32.5%)	
	Winter	480 (23.5%)		34(18.7%)		104(23%)		9(22.5%)	
	*p* value	0.007		0.254		0.337		0.572	
	Season at onset of DM^*a*^								
	Spring	506(24.8%)		42(23.1%)		114(25.2%)		11(27.5%)	
	Summer	364(17.8%)		34(18.7%)		89(19.6%)		9(22.5%)	
	Autumn	542 (26.5%)		49(26.9%)		142(31.3%)		12(30%)	
	Winter	631(30.9%)		57(31.3%)		108(23.8%)		8(20%)	
	*p* value	< 0.001		0.026		0.005		0.801	

Table 2 shows the season of birth and season of diagnosis according to the participants’ age at DM onset and the number of autoimmune diseases.*n* number, *DM* Diabetes Mellitus, *HT* Hashimoto thyroiditis, *CD* Celiac disease. Continuous variables were expressed as mean ± standard deviation (SD), while categorical variables were presented as frequencies and within-group percentages. mean ± SD, n (%). ^*a*^*p* < *.05 assessed through ****chi-squared analyses ***^***b***^*p* < *.05 assessed through ****independent samples t- analysis***

### Seasonality by birth and diagnosis

Patients with only T1DM were more frequently born in spring–summer (*p* = 0.001), but this pattern was limited to those diagnosed after age 3 (*p* = 0.007). No birth season differences were seen in patients with autoimmune comorbidities. Table 2 shows the birth season and season of diagnosis according to the participants’ age at DM onset and the number of autoimmune diseases.

Seasonality in diagnosis was evident among patients with T1DM only (*p* < 0.001), DM + CD (*p* = 0.025), and DM + HT (*p* = 0.012), with peaks in autumn–winter (Table 2). However, no seasonal trend was found in early-onset (< 3 years) or multiple-autoimmunity groups, supporting a predominant role for intrinsic immunological mechanisms.

Multivariable binary logistic regression analyses were performed separately for patients with diabetes onset before 3 years of age and those with onset at 3 years of age or older to evaluate the independent associations of season at diabetes onset, birth season, and gender with the presence of concomitant autoimmune disease (Table 3). The dependent variable was the presence of at least one concomitant autoimmune disease. The overall model was statistically significant (χ^2^ = 48.525, *p* < 0.001).

**Table 3:** Multivariable logistic regression analysis of factors associated with concomitant autoimmune disease according to age at onset of DM

Variables	Age at onset < 3						Age at onset ≥ 3					
	*B*	SE	*p*	Exp (B) (OR)	%95 CI		*B*	SE	*p*	Exp (B) (OR)	%95 CI	
					Lower	Upper					Lower	Upper
Season at onset of DM	−0,085	0,109	0,434	0,919	0.742	1,137	0,087	0,040	**0,029**	1,090	1,009	1,179
Season at birth	−0,045	0,114	0,691	0,956	0,764	1,195	0,007	0,043	0,878	1,007	0,926	1,094
Gender (F/M)	0,647	0,245	**0,008**	1,909	1,181	3,086	0,612	0,094	** < 0,001**	1,844	1,535	2,215

*DM* diabetes mellitus; *B* regression coefficient, ***SE*** standard error, *p* p value, *Exp(B)* exponentiated B coefficient/odds ratio, *OR* odds ratio, *CI* confidence interval. Gender was coded as female/male

Among patients with diabetes onset before 3 years of age, season at diabetes onset and birth season were not significant predictors (*p* > 0.05). However, among patients with diabetes onset at ≥ 3 years of age, season at diabetes onset showed a weak but significant association with concomitant autoimmune disease (OR = 1.090, 95% CI: 1.009–1.179, *p* = 0.029). Gender remained significantly associated with concomitant autoimmune disease, with higher odds observed in female patients (OR = 1.844, 95% CI: 1.535–2.215, *p* < 0.001) (Table 3).

### Summary of statistical trends

Early-onset T1DM: no seasonal trend (*p* > 0.05).

Late-onset T1DM: autumn–winter peak (*p* < 0.001).

DM + CD + HT group: absence of seasonal effect (*p* = 0.801).

These data reveal a gradient from environmental to immunological dominance as autoimmunity intensifies.

## Discussion

Type 1 DM incidence continues to rise globally, increasing by 3–5% annually [15]. Italy ranks 12th globally in terms of the highest number of diabetes cases based on the number of affected individuals [16]. The rapid rise in the incidence of DM worldwide, including in Italy, may be attributed to growing environmental factors acting upon a common genetic susceptibility. In the Nationwide Italian Twin Study, which we previously conducted with the ISPED study group, we demonstrated a significant and substantial role of shared environmental factors, particularly during intrauterine or early postnatal life, in the susceptibility to T1DM and its increasing incidence [15]. Based on these data, it is estimated that avoiding or eliminating these exogenous factors could prevent the rising incidence of the disease. Therefore, in this 40-year, single-center retrospective cohort study, we evaluated the effects of genetic and environmental factors in early and late-onset diabetes. Our study provides one of the most comprehensive analyses to date on the etiological interaction between immune dysregulation and environmental seasonality in T1DM. Our findings suggest that the contribution of environmental factors to the seasonal pattern of T1DM may differ according to age at onset and autoimmune comorbidity. Specifically, seasonality appeared to be attenuated in children diagnosed before the age of 3 years and in those with multiple autoimmune diseases, suggesting the possibility of differing etiological pathways within the spectrum of childhood T1DM.

The mild male predominance observed in late-onset T1DM aligns with prior epidemiologic studies showing a 1:1 to 2:1 male-to-female ratio [17–20]. This pattern contrasts with the female bias typically observed in HT [18]. Some studies have indicated that CD is more comon in women or no significant gender-related difference was found for this disease [18, 21, 22]. These differences may reflect sex-specific variations in immune cell activation, hormonal regulation, and insulin sensitivity**.** The higher prevalence of Hashimoto’s thyroiditis among girls, especially those with longer T1DM duration, also mirrors established autoimmune clustering patterns. The strong association between early age of T1DM onset and celiac disease supports the concept that autoimmune aggregation reflects an underlying immunogenetic vulnerability rather than coincidental comorbidity**.**

Patients born in spring–summer months were more frequently diagnosed with T1DM after the age of three. Among these, July accounted for the highest proportion of births (10.2%), followed by June and August (8.9%). This observation supports the “critical window” hypothesis, suggesting that early developmental exposures may influence later disease risk.

The relationship between birth month and T1DM onset likely reflects complex interactions between climatic factors and maternal sunlight exposure during prenatal pancreatic β cell development, as well as the infant’s postnatal exposure during pancreatic maturation period. One possible explanation proposed in previous studies is that conception during autumn and winter may increase fetal exposure to maternal infections, nutritional deficiencies or reduced ultraviolet exposure [23–25]. In their extensive study of 1118 children, 810 adolescents, and young adults diagnosed with DM, Songini et al. found a higher incidence of DM in those born in summer months [17]. Although our findings are compatible with this hypothesis, our study was not designed to evaluate these environmental exposures directly.

Fetal pancreatic organogenesis begins early in gestation: the pancreas becomes identifiable by the fourth week, while beta-cell development occurs between the eighth and ninth weeks [26]. Intrauterine pancreatic programming is known to be influenced by maternal stressors such as infections, malnutrition, smoking, and alcohol consumption [27]. The higher prevalence of T1DM among children born in summer therefore supports the hypothesis that fetuses conceived during infection-prone autumn–winter months may be exposed to maternal immune activation, potentially disrupting endocrine pancreatic development and predisposing to future β-cell autoimmunity.

The cut-off age of three years was chosen because children under three typically have limited outdoor exposure. Because children younger than 3 years generally have lower exposure to daycare environments and peer-related infectious contacts [12]**.** We hypothesized that environmental influences might be less pronounced in this age group. Consistent with this hypothesis, we observed no significant seasonal pattern among children diagnosed before the age of 3 years. However, because genetic susceptibility, HLA status, autoantibody profiles and other immunological markers were not evaluated, these findings should be interpreted as indirect evidence rather than proof of predominant genetic mechanisms.

Late-onset T1DM showed a marked seasonal pattern, peaking during autumn and winter. These findings are consistent with previous reports suggesting a possible contribution of environmental exposures such as viral infections, reduced sunlight exposure, and lower vitamin D levels [5–7, 28, 29]. The predominance of T1DM diagnoses in colder months may additionally reflect behavioral and metabolic changes during winter**,** including reduced physical activity, altered dietary intake, increased respiratory infections, and higher air pollution exposure [30–32], all potential contributors to immune activation and insulin resistance. Such factors likely interact with seasonal viral infections (e.g., enterovirus, rotavirus, rubella) that have been repeatedly linked to islet cell autoimmunity [33]. In contrast, the absence of seasonality in early-onset (< 3 years) suggests that strong intrinsic immunogenetic mechanisms dominate before significant environmental exposure occurs. These children likely experience accelerated β-cell autoimmunity independent of seasonal factors.

To assess whether other coexisting autoimmune diseases impact the seasonal pattern of the disease, patients were categorized into four groups: T1DM only, T1DM + CD, T1DM + HT, and T1DM + CD + HT, and analyzed separately. An important finding of our study was the attenuation of seasonality among patients with multiple autoimmune diseases (T1DM + CD + HT). One possible explanation is that individuals with a greater burden of autoimmune disease may represent a subgroup with stronger underlying immunological susceptibility, resulting from the continuous stimulation of mediated T-lymphocytes [34]. This may override external triggers, leading to disease onset regardless of season.

Previous studies have emphasized the importance of using etiological rather than clinical criteria to define T1DM, as this approach allows for a better understanding of its epidemiology and natural history of the disease [35, 36]. None of the earlier studies has considered the number of coexisting autoimmune diseases and their relationship with the seasonality of T1DM. Key strengths of this study include its large, well-characterized cohort**,** uniform diagnostic approach, and continuous follow-up by the same clinical team for four decades, minimizing classification bias. Moreover, by stratifying patients according to both age at onset and autoimmune burden**,** this study provides novel insight into how immune intensity modulates environmental susceptibility—an aspect rarely addressed in previous literature. By excluding congenital diabetes, which differs from T1DM primarily in its potential for treatment with hypoglycemic agents and its significantly lower frequency of chronic complications [37], as well as other diseases that progress with hyperglycemia, such as MODY and T2DM, we were able to provide stronger evidence for the etiology of T1DM.

Limitations include the retrospective design and the inability to directly assess environmental exposures (e.g., infections, vitamin D status). Another important limitation is that no genetic or immunological data (including HLA genotypes, islet autoantibodies, or immune biomarkers) were available. Consequently, although the observed attenuation of seasonality in early-onset and multiple-autoimmune cases is compatible with a stronger genetic or immunological contribution, this hypothesis cannot be confirmed directly by our data. Although diagnostic criteria evolved over the 40-year period, retrospective re-evaluation according to current ISPAD standards mitigated potential misclassification. Finally, the date of diagnosis may not perfectly correspond to disease onset due to preclinical progression, a limitation inherent to all retrospective studies. Future population-based screening for islet autoantibodies will allow earlier detection and more accurate temporal mapping of environmental triggers.

### Clinical and research ımplications

Our results emphasize that T1DM is not a single disease entity but rather a spectrum with distinct etiological subtypes. Early-onset and multi-autoimmune forms appear to represent genetically “hard-wired” autoimmunity**,** whereas late-onset T1DM reflects environmentally modulated immune activation. This distinction has important implications for prevention: public-health strategies targeting infection control, vitamin D sufficiency, and early immune monitoring may be most effective in populations at risk for late-onset disease. Conversely, genetic screening and immune-profiling could improve early detection in high-risk families.

## Conclusions

In summary, this long-term cohort provides compelling evidence that the interaction between an increasing number of autoimmune diseases and environmental seasonality shapes T1DM onset. Environmental triggers predominate in later-onset cases, while robust genetic autoimmunity drives early disease and multi-autoimmune presentations. Recognizing this heterogeneity is essential and should be taken into account for designing age- and immunity-specific screening and prevention strategies, ultimately paving the way for personalized approaches to Type 1 diabetes prevention.

## Data Availability

Data available on request from the corresponding author.
